# Unique metabolic features of pancreatic cancer stroma: relevance to the tumor compartment, prognosis, and invasive potential

**DOI:** 10.18632/oncotarget.11893

**Published:** 2016-09-07

**Authors:** Erik S. Knudsen, Uthra Balaji, Elizaveta Freinkman, Peter McCue, Agnieszka K. Witkiewicz

**Affiliations:** ^1^ McDermott Center for Growth and Development, University of Texas Southwestern Medical Center, Dallas, TX, USA; ^2^ Simmons Cancer Center, University of Texas Southwestern Medical Center, Dallas, TX, USA; ^3^ University of Arizona Cancer Center, University of Arizona, Tucson, AZ, USA; ^4^ Department of Medicine, University of Arizona, Tucson, AZ, USA; ^5^ Whitehead Institute, Massachusetts Institute of Technology, Cambridge, MA, USA; ^6^ Department of Pathology, Thomas Jefferson University, Philadelphia, PA, USA; ^7^ Department of Pathology, University of Arizona, Tucson, AZ, USA

## Abstract

Pancreatic ductal adenocarcinoma (PDAC) has a dismal prognosis. The aggressiveness and therapeutic recalcitrance of this malignancy has been attributed to multiple factors including the influence of an active desmoplastic stroma. How the stromal microenvironment of PDAC contributes to the fatal nature of this disease is not well defined. In the analysis of clinical specimens, we observed diverse expression of the hypoxic marker carbonic anhydrase IX and the lactate transporter MCT4 in the stromal compartment. These stromal features were associated with the epithelial to mesenchymal phenotype in PDAC tumor cells, and with shorter patient survival. Cultured cancer-associated fibroblasts (CAFs) derived from primary PDAC exhibited a high basal level of hypoxia inducible factor 1a (HIF1α) that was both required and sufficient to modulate the expression of MCT4. This event was associated with increased transcription and protein synthesis of HIF1α in CAFs relative to PDAC cell lines, while surprisingly the protein turnover rate was equivalent. CAFs utilized glucose predominantly for glycolytic intermediates, whereas glutamine was the preferred metabolite for the TCA cycle. Unlike PDAC cell lines, CAFs were resistant to glucose withdrawal but sensitive to glutamine depletion. Consistent with the lack of reliance on glucose, CAFs could survive the acute depletion of MCT4. In co-culture and xenograft studies CAFs stimulated the invasive potential and metastatic spread of PDAC cell lines through a mechanism dependent on HIF1α and MCT4. Together, these data indicate that stromal metabolic features influence PDAC tumor cells to promote invasiveness and metastatic potential and associate with poor outcome in patients with PDAC.

## INTRODUCTION

Pancreatic ductal adenocarcinoma (PDAC) has a poor prognosis with a 5 year survival rate of less than 6%[1–3]. Even in patients with curative resection, disease recurrence occurs in the majority of cases. Systemic therapies have modest durable impact [4–6]; therefore, PDAC represents a therapy-recalcitrant disease for which new approaches to treatment are urgently needed. It is established that growth of tumor cells is supported by oncogenic signaling and altered metabolism [7–9]. Currently, it is largely unknown whether the metabolic wiring of cancer is induced exclusively by driver mutations or is a manifestation of selection in a particular tumor microenvironment. Understanding the metabolic features of PDAC, key mediators, and related dependencies could yield important insights into the pathogenesis of disease and new targets for tumor therapy. Aberrant metabolism is considered one of the hallmarks of cancer. It has been demonstrated that KRAS and TP53 mutations are key determinants of the PDAC metabolic state [10–13]. In this context, studies have shown that PDAC exhibits increased glycolysis and concomitant use of glucose intermediates into the hexosamine and pentose phosphate pathways. Other groups have suggested that PDAC is dependent on glutamine metabolism and that inhibition of macropinocytosis and related scavenging of amino acids suppresses pancreatic cancer growth in xenograft models [14–16]. A recent study indicated that there are specific metabolic subtypes of PDAC, and that metabolic state impacts on vulnerabilities to metabolic challenges and therapy [17].

The drivers of PDAC metabolic preference are likely reflective of both oncogenic signaling pathways and the unique desmoplastic stromal microenvironment [13, 18–20]. It has been proposed that poor vascularization and hypoxia contribute to metabolic features of PDAC, while others have recently demonstrated that stromal diversity (activated *vs*. normal stroma) impacts on PDAC biology and prognosis [19, 21–24]. The desmoplastic stroma often accounts for more than 50% of the tumor mass, and this feature of disease is believed to contribute to PDAC pathogenesis and therapeutic resistance [22, 24–26]. Also, it has been shown that in some instances the stroma can constrain rampant tumorigenic growth [27, 28]. Since the stromal compartment accounts for the majority of PDAC volume, its significance to PDAC metabolism is crucial. Furthermore, it is recognized that targeting signaling pathways activated in the stroma might be necessary to increase the efficacy of PDAC therapy [22, 26, 29]. Surprisingly, relatively few analyses have delineated metabolic features of PDAC stroma and its impact on tumor cell biology.

The hypoxic environment of PDAC and other tumor types is believed to contribute to the preference for glycolysis, with many cancer cells converting the majority of glucose into lactate. This metabolic preference is maintained even in the presence of sufficient oxygen [30, 31]. Glycolysis, although generating a low yield of ATP per glucose molecule consumed, is the pathway of choice for rapid cell division because it generates intermediates for anabolic reactions [16], and is less prone to generating reactive oxygen species (ROS). To adapt to increased production of lactate, cancer cells utilize various regulation systems including carbonic anhydrase IX (CAIX) and proton-coupled monocarboxylate transporters (MCTs) [32–34]. CAIX buffers the extracellular environment by converting bicarbonate and carbon dioxide. In contrast, MCT proteins modulate the extracellular pH through proton-coupled metabolite transport. MCT4 has generally been ascribed to exclusively mediate lactate efflux and lower extracellular pH. Both CAIX and MCT4 are targets of the HIF1α transcription factor and their expression has been postulated to denote hypoxic tumors [35, 36]. MCT4 is highly expressed in PDAC, associates with poor prognosis and glycolytic metabolism, and is required for glycolytic flux in cell line models [18]. In tumor cells with high levels of MCT4, there is an “addiction” to the transporter, wherein depletion leads to multiple metabolic adaptations, but tumor cell death occurs rapidly and MCT4 depletion limits tumorigenic growth in xenograft models [18]. Here we used a combination of primary PDAC tissue specimens, patient-derived cancer associated fibroblasts (CAFs), and extensive metabolomics analyses to delineate the metabolic features of PDAC stroma and symbiotic effects on the tumor compartment mediated by HIF1α and MCT4.

## RESULTS

### Hypoxia markers in the tumor microenvironment are potent determinants of PDAC prognosis

**Figure 1:**
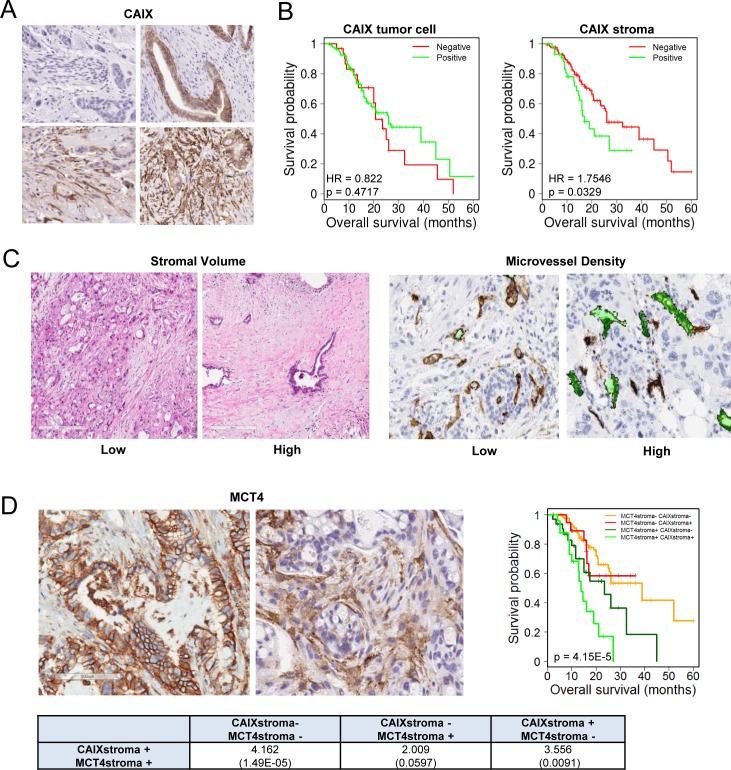
CAIX expression in PDAC and association with outcome **A.** Representative images of immunohistochemical staining showing: negative expression of CAIX in both tumor cell and stromal compartments (top left), positive expression of CAIX in tumor cells but not stromal compartment (top right), positive expression of CAIX in stromal compartment but not tumor cells (bottom left), positive expression of CAIX in both tumor cell and stromal compartments (bottom right). **B.** Kaplan-Meier analysis of CAIX expression in the distinct tumor cell and stromal compartments and the associated overall survival. **C.** Representative images of immunohistochemical staining showing low stromal volume and high stromal volume and low and high microvessel density **D.** Representative images of immunohistochemical staining showing: positive expression of MCT4 in tumor cell compartment and positive expression of MCT4 in tumor stromal compartment. Kaplan-Meier analysis of combinatorial MCT4 and CAIX expression in the stromal compartment and the associated overall survival. Hazard ratios and *p*-values for each comparison are summarized in the table.

**Figure 2:**
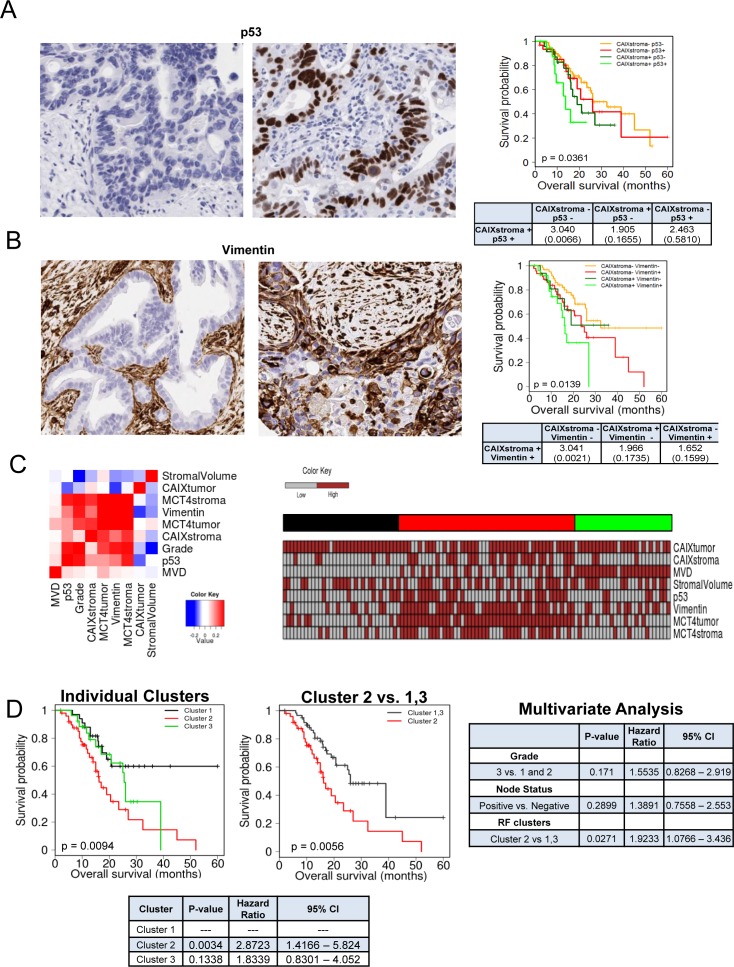
Subtypes of PDAC stroma are associated with distinct tumor features to delineate prognosis **A.** Representative images of immunohistochemical staining showing: negative expression of TP53 in tumor cell compartment (left), positive expression of TP53 in tumor cell compartment (right). Kaplan-Meier analysis of combinatorial TP53 and stromal CAIX expression and the associated overall survival. Hazard ratios and *p*-values for each comparison are summarized in the table. **B.** Representative images of immunohistochemical staining showing: negative expression of vimentin in tumor cell compartment (left), positive expression of vimentin in tumor cell compartment (right). Kaplan-Meier analysis of combinatorial vimentin and stromal CAIX expression and the associated overall survival. Hazard ratios and *p*-values for each comparison are summarized in the table. **C.** Correlation heatmap for the indicated markers stained in the PDAC cohort. Heatmap demonstrating the expression of all markers investigated and resulting random forest clusters. **D.** Kaplan-Meier analysis of individual random forest clusters and their association with survival (left), and Kaplan-Meier analysis of cluster 2 compared to cluster 1 and 3 and their association with survival (center). Multivariate analysis of tumor grade, nodal status and random forest cluster (right). Hazard ratios and *p*-values for each comparison are summarized in the table (bottom).

One of the unique features of PDAC is the presence of abundant desmoplastic stroma, which has been implicated in inducing a hypoxic state by constraining tumor vasculature. To determine the relationship between hypoxia and the tumor stroma, we utilized a patient cohort of 203 PDAC cases (demographics, Supplemental Table 1). We found that the marker of hypoxia carbonic anhydrase IX (CAIX) was expressed in the stromal environment of PDAC (Figure 1A), and interestingly was not significantly associated with expression in the tumor cells (p = 0.085, Supplemental Table 2). CAIX expression in the stromal compartment was associated with poor prognosis, while the expression in the tumor cells only trended toward poor outcome (Figure 1B). Surprisingly, stromal expression of CAIX was independent of high stromal volume and low microvessel density (Figure 1C, Supplemental Table 3), but was associated with stromal expression of another hypoxia marker MCT4 (Figure 1D, Supplemental Table 3). Tumors with high levels of CAIX and MCT4 in the stroma had particularly poor prognosis (Figure 1D). To determine potential features of the tumor that would be related to having high levels of CAIX/MCT4 in the stromal compartment, multiple additional markers were analyzed. PDAC with high stromal CAIX/MCT4 expression were characterized by presence of high levels of p53 protein, which is indicative of TP53 mutations. Additionally, many such tumors exhibited evidence of the epithelial to mesenchymal transition (EMT) phenotype as determined by the expression of vimentin in the tumor cells (Figure 2A and 2B, Supplemental Table 3). Pearson correlation revealed that TP53 mutations and EMT were highly correlated with MCT4 and CAIX expression in the tumor stromal environment (Figure 2C). Random forest clustering of the markers partitioned PDAC cases into discrete clusters (Figure 2D). In this context, cluster 2 characterized by stromal expression of MCT4 and CAIX, was associated with particularly poor prognosis and that finding remained significant in multivariateanalysis (Figure 2E). In contrast, cluster 1 encompassing poorly vascularized tumors with the absence of CAIX and MCT4 in the stroma, had a better prognosis. These data suggest that stromal features of disease are associated with survival in PDAC.

### CAFs exhibit elevated HIF1α and MCT4

**Figure 3:**
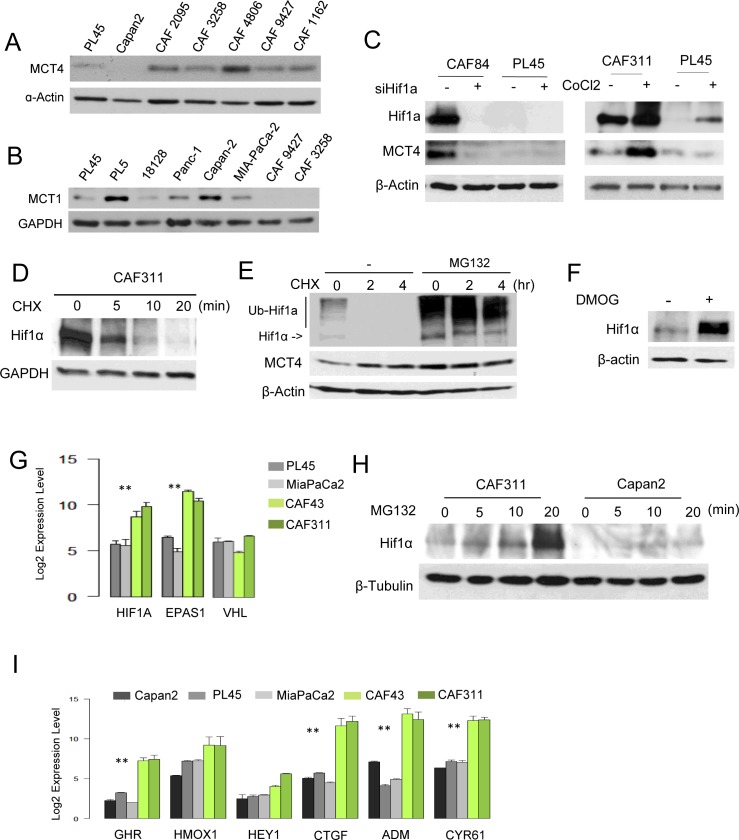
Aberrant HIF1α expression in PDAC cancer associated fibroblasts **A.** The level of MCT4 was determined in the indicated established pancreatic cancer cell lines and CAF cultures by immunoblotting. **B.** The level of MCT1 was determined in the indicated established pancreatic cancer cell lines and CAF cultures by immunoblotting. **C.** HIFα and MCT4 levels were determined by immunoblotting following the knockdown of HIF1α with RNAi or the induction of hypoxia through the use of CoCl_2_. **D.** The stability of HIF1α was evaluated in CAFs following treatment with cycloheximide. **E.** HIF1α was detected by immunoblotting in the presence of cycloheximide in the absence or presence of the proteasome inhibitor MG132. **F.** HIF1α protein levels were determined in the absence and presence of DMOG. **G.** RNA levels of the indicated genes as determined by microarray analyses (**p < 0.01 for tumor cell line *vs*. CAF). **H.** MG132 was used to interrogate the synthetic rate of HIF1α protein in the indicated cell lines. **I.** The expression of HIF1α target genes was evaluated in the indicated cell lines by microarray analysis (**p < 0.01 for tumor cell *vs*. CAF).

In order to differentiate the compartment selective roles of hypoxia markers, we employed CAFs cultures that were isolated from multiple PDAC specimens. Analysis of CAFs cultures revealed diversity in the expression of MCT4, with a majority of the cultures expressing high levels of MCT4 protein and RNA relative to PDAC tumor cell lines (Figure 3A, Figure S1). CAFs did not express MCT1 (Figure 3B) and this finding was consistent with the lack of stromal MCT1 staining in PDAC clinical cases [18]. Interestingly, CAFs expressing high levels of MCT4 also harbored exceedingly high levels of HIF1α (Figure 3C). Knockdown studies showed that HIF1α was required for the expression of MCT4, and even the high basal levels of HIF1α could be further stimulated with CoCl_2_ and, in turn, lead to further induction of MCT4 (Figure 3C). Protein stability is typically the most relevant determinant of HIF1α accumulation; however, HIF1α half-life was approximately 5 minutes in CAFs (Figure 3D). Furthermore, treatment with either proteasome inhibitors or inhibitors of prolyl hydroxylation comparably stabilized HIF1α (Figure 3E and 3F). These data suggest that the basis for the increased HIF1α protein levels in pancreatic cancer CAFs is not necessarily a reflection of deficits in protein turnover. Rather, CAFs exhibited significantly elevated levels of the HIF1α transcript (Figure 3G). Correspondingly, with short exposure to proteasome inhibitor MG132 the increase in HIF1α occurred rapidly in CAFs relative to tumor cell lines (Figure 3H). Consistent with these findings, CAFs were characterized by having elevated basal expression of multiple HIF1α target genes (Figure 3I). Together, these data demonstrate that high-levels of HIF1α/MCT4 in CAFs represent a programmed event.

### CAFs employ glutamine as a key energy source

**Figure 4:**
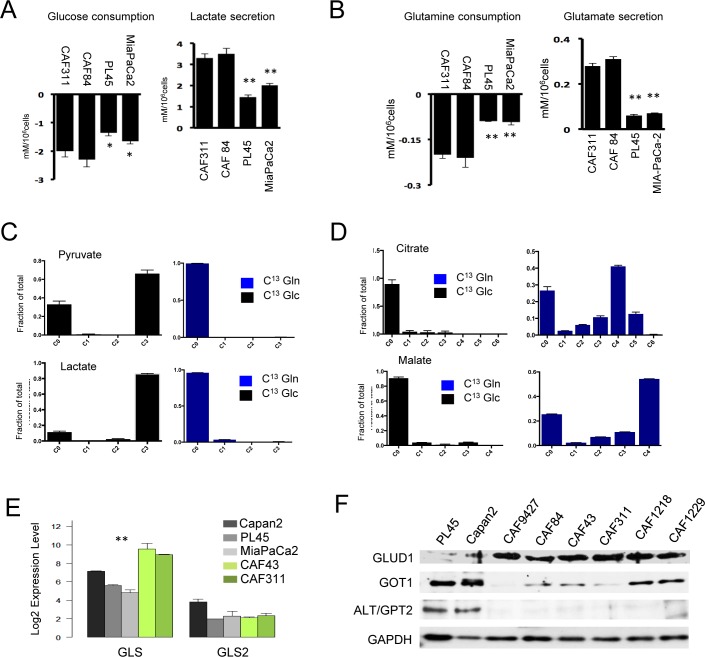
Unique metabolic features of cancer associated fibroblast cultures **A.**/**B.** The media from CAF cultures and PDAC cell lines were analyzed for glucose and glutamine consumption and the production of glutamate and lactate (*p < 0.05 or **p < 0.01 for tumor cell line *vs*. CAF). **C.** Cells were labeled with U^13^C-Glucose or U^13^C-Glutamine and the fractional mass-labeling of carbons in pyruvate and lactate are shown. **D.** Cells were labeled with U^13^C-Glucose or U^13^C-Glutamine and the fractional mass-labeling of carbons in malate and citrate are shown. **E.** The indicated genes associated with glutamine metabolism were evaluated in the indicated cell lines by microarray analysis (**p < 0.01 for tumor cell line *vs*. CAF). **F.** The indicated proteins associated with glutamine metabolism were evaluated in the indicated cell lines by immunoblot analysis.

Since CAFs express very high levels of HIF1α, it would suggest that they utilize predominantly glycolytic metabolism. Analysis of metabolic features of the cells by surveying consumption and excretion of metabolites showed that CAFs were active for the uptake of glucose and glutamine relative to the glycolytic cancer cell lines PL45 and MiaPaCa2 (Figure 4A and 4B). Correspondingly, they secreted increased levels of both glutamate and lactate. In order to delineate the predominant utilization of glucose and glutamine, CAFs were labeled with U^13^C-glucose and U^13^C-glutamine. The data indicated that, as expected, glucose was the predominant carbon source for glycolysis (Figure 4C). In contrast, glutamine was efficiently metabolized to TCA intermediates (Figure 4D). The synthesis of ^13^C_4_-succinate, -fumarate, and -malate (m+4) was consistent with the oxidation of glutamine derived ^13^C_5_-α ketoglutarate via the forward reactions of the TCA cycle. We also observed evidence of reductive carboxylation to form ^13^C-citrate from the glutamine tracer. However, the relative distributions of the m+5 versus m+3, m+4, and m+6 mass isotopomers, which are characteristic of the forward reactions in the TCA plus pyruvate carboxylase activity, indicated that reductive carboxylation was not the major pathway. Analysis of key determinants of glutamine metabolism indicated that CAFs expressed high levels of glutaminase (GLS1) and glutamate dehydrogenase (GLUD1) that mobilized the metabolism of glutamine in the TCA cycle (Figure 4E and 4F). In contrasts to PDAC cells, CAFs expressed low levels of cytosolic aspartate transaminase (GOT1) and alanine transaminase (ALT/GPT2) indicating that each cell type has unique metabolic adaptations.

**Figure 5:**
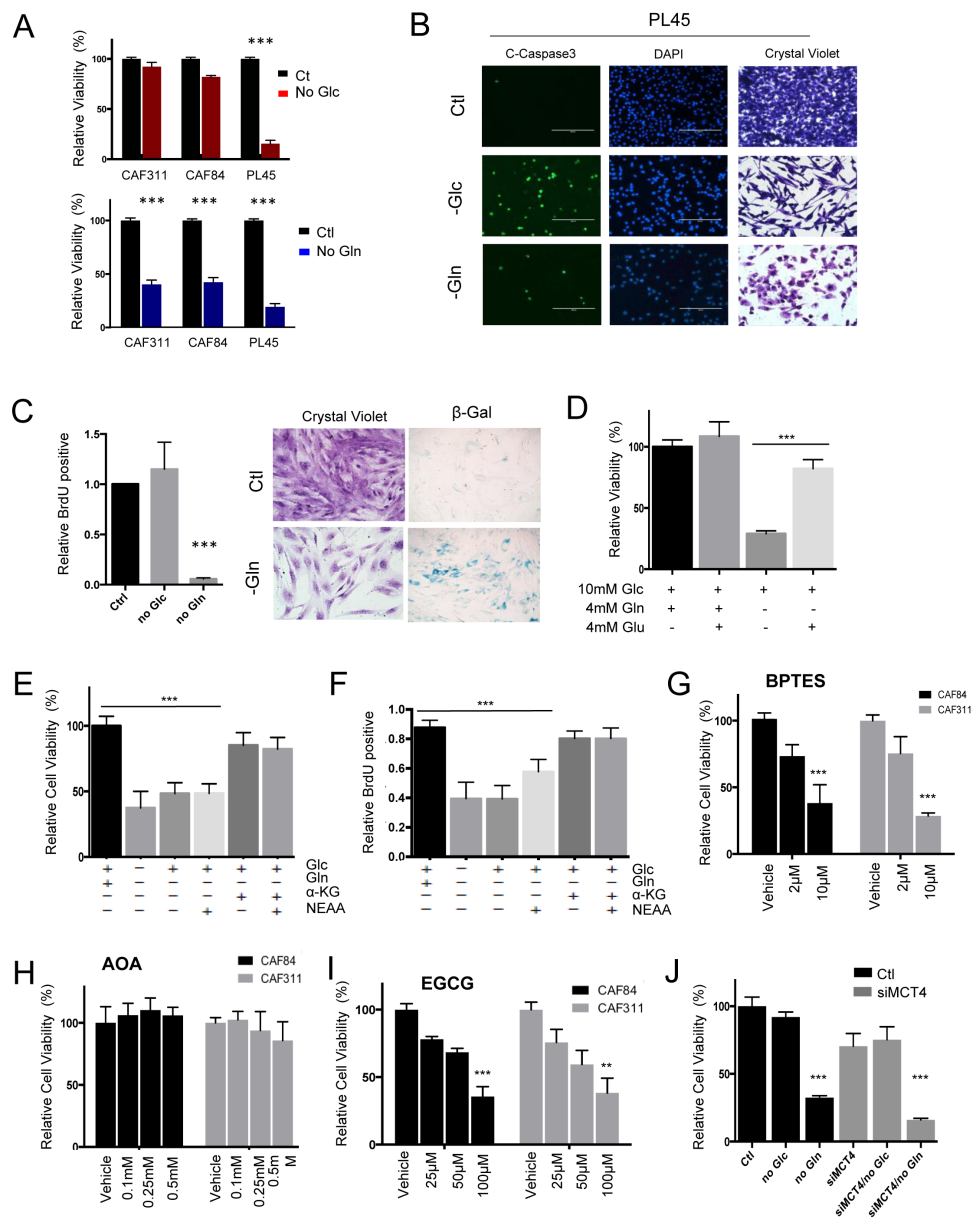
Unique metabolic dependences of cancer associated fibroblasts **A.** The dependence of CAFs and tumor cell lines on glucose or glutamine was evaluated by nutrient withdrawal from the media (***p < 0.001). **B.** The identification of apoptosis in tumor cell lines was determined through the use of cleaved caspase 3. **C.** The impact of glutamine and glucose withdrawal in CAFs was determined by BrdU incorporation, crystal violet staining, and staining for senescence-associated ß-galactosidase activity (***p < 0.001). **D.** The influence of the indicated metabolites on CAF viability was determined (Glc-glucose, Gln-glutamine, Glu-glutamate). **E.**/**F.** The influence of the indicated metabolites on CAF viability or BrdU incorporation was determined (Glc-glucose, Gln-glutamine, aKG- α-ketoglutarate, NEAA-non essential amino acids) (***p < 0.001 relative to the control). **G.** The influence of the GLS inhibitor BPTES on cell viability of CAFs (***p < 0.001 relative to the control). **H.** The influence of aminotransferase inhibitor aminooxyacetate (AOA) on cell viability of CAFs. **I.** The influence of GLUD1 inhibitor EGCG on CAFs viability. **J.** The impact of MCT4 knockdown on cell viability was evaluated in CAFs showing minimal impact on cell viability (***p < 0.001 relative to the control).

In order to assess the functional significance of metabolite utilization, CAFs and tumor cell lines were depleted for glucose or glutamine (Figure 5A and S2). These data showed that tumor cells (e.g. PL45) were very sensitive to depletion of either metabolite, which is consistent with previously published studies [10, 18]. However, CAFs were selectively sensitive to glutamine withdrawal and resistant to the depletion of glucose (Figure 5A and S2). This observation was recapitulated in co-culture, wherein glucose withdrawal would selectively elicit killing of the tumor cells (Figure S2). Interestingly, while nutrient withdrawal resulted in cell death in cell lines (Figure 5B), glutamine withdrawal in CAFs was accompanied by cell cycle inhibition and induction of senescence (Figure 5C). The impact of glutamine withdrawal, in CAFs could be rescued with glutamate (Figure 5D) and α-ketoglutarate, as expected for utilization of glutamine within the mitochondria (Figure 5E). Importantly, the addition of α-ketoglutarate also restored the proliferative capacity of the CAFs (Figure 5F). Consistent with the mitochondrial utilization of glutamine, the GLS1 and GLUD1 inhibitors BPTES (Figure 5G) and EGCG (Figure 5I) inhibited the growth of CAFs, whilst the transaminase inhibitor AOA (Figure 5H) had no effect on CAFs. Since, unlike tumor cells, CAFs do not appear to be reliant on glucose metabolism for survival, there was not a significant impact of MCT4 depletion on the viability of CAFs in spite of the exceedingly high MCT4 levels present in the stromal cells (Figure 5J and S2).

### CAFs secrete multiple metabolites that could fuel tumor cell proliferation

**Figure 6:**
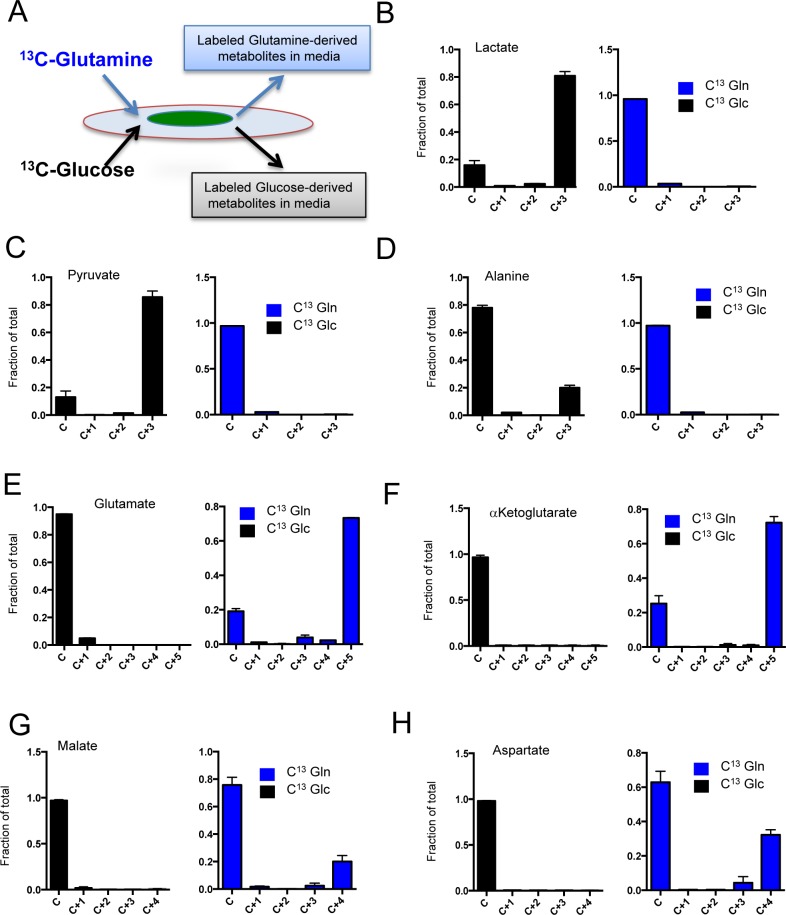
Metabolite secretion from CAFs **A.** In order to determine the specific generation of metabolites from CAFs to the tumor environment, cells were labeled with U^13^C-Glucose or U^13^C-Glutamine and the metabolites in the media were evaluated by mass-spectrometry as shown in the schematic. **B.**/**H.** The fractional mass-labeling of metabolites in the media as determined from cells labeled with U^13^C-Glucose (black bars) or U^13^C-Glutamine (blue bars).

It is recognized that CAFs contribute to the biology of PDAC through multifactorial mechanisms. Here, we focused on determining which metabolites could be produced by CAFs and then secreted into the local environment to impact tumor cells. For these studies, two different CAF cultures with high MCT4 and HIF1α expression were labeled with U^13^C-glucose and U^13^C-glutamine, and media was analyzed for the presence of labeled intermediates that by definition had to be derived from the CAFs (Figure 6A). These analyses showed that veritably all lactate and pyruvate recovered from the media arose from glucose metabolism in the CAFs (Figure 6B and 6C). Interestingly, of over 20 additional metabolites analyzed, only alanine in the media could be traced to glucose metabolism in the CAFs (Figure 6D). Glutamine metabolism was responsible for the majority of the extracellular pool of glutamate (Figure 6E) and α-ketoglutarate (Figure 6F). Glutamine-derived aspartate and malate were also detected in media; however, this represented a more modest fraction of the extracellular pool of these metabolites (Figure 6G and 6H). Together, these data indicated that a surprising diversity of metabolites were shed by CAFs and in principle could impact the biology of the surrounding tumor.

### MCT4 is associated with extracellular 3-carbon metabolite pools and drives the invasive features of PDAC tumors

**Figure 7:**
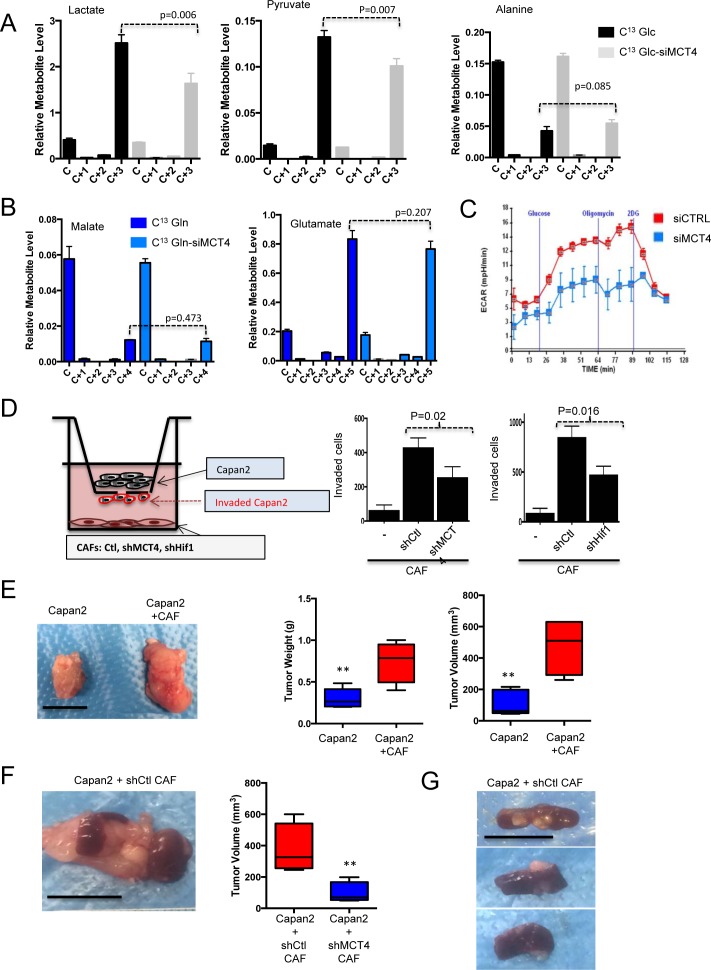
Metabolic features of CAFs support tumor cell invasion/metastasis **A.** The effect of MCT4 depletion was evaluated on the secretion of glucose-derived metabolites. Only the levels of lactate and pyruvate in the media were reduced with MCT4 knockdown. **B.** The effect of MCT4 depletion was evaluated on the secretion of glutamine-derived metabolites. There was no significant effect on any metabolite (glutamate and malate are shown). **C.** MCT4 knockdown limits glycolytic activity as measured by ECAR and acidification of the media. **D.** The invasive potential of Capan2 tumor cells was evaluated in modified Boyden chamber assays. CAFs were plated at the bottom of the wells and invasion was quantified. Data showed that CAFs supported invasion, which was dependent on the expression of HIF1α and MCT4 in the CAF. **E.** Co-injection of CAFs with Capan2 was evaluated in subcutaneous xenograft models. Impact on tumor volume and tumor mass was determined. **F.** Orthotopic co-injection was performed with CAFs and Capan2. Tumor mass and weight was significantly increased with MCT4 expression. **G.** Representative spleen metastasis observed with CAFs expressing MCT4.

MCT4 knockdown was used to determine the effect on extracellular metabolite pools derived from CAF metabolism. These data revealed that MCT4 depletion limited the production of extracellular labeled lactate and pyruvate from CAFs (Figure 7A). In contrast, there was no effect on extracellular glucose-derived alanine or on glutamine-derived metabolites (Figure 7B). Consistent with these findings, there was a suppression of media acidification as measured by ECAR analysis (Figure 7C). To determine if secreted metabolites impact tumor cells, Boyden chamber assays were employed (Figure 7D). In this context, MCT4 high CAFs stimulated the invasion of PDAC cells. Knockdown of MCT4 limited the invasive potential, as did the knockdown of HIF1α (Figure 7D). Similar results were also observed using CAFs that exhibit intrinsically different levels of HIF1α/MCT4 (Figure S3). In co-culture wound healing assays, CAFs supported invasion of the tumor cells in a manner similarly dependent on the presence of MCT4 (Figure S3). To determine whether these events were relevant in the context of tumorigenic capacity *in vivo*, subcutaneous xenograft models were employed, where Capan2 cells were injected alone or with CAFs that expressed high levels of MCT4. These CAFs significantly stimulated tumor growth (Figure 7E and 7F) and lead to formation of tumors that were locally invasive into the skeletal muscle (Figure S4). To better delineate the specific role of MCT4, shRNA mediated knockdown was employed in CAFs and they were orthotopically co-injected with Capan2 cells in the pancreas of NSG mice (Figure 7G). In this context, the presence of MCT4 expressing CAFs resulted in larger tumor size and presence of visceral metastasis to the spleen and liver (Figure 7H). Metastases were not observed in the absence of CAFs, and were diminished with MCT4 knockdown in the CAFs (Figure S4). Together these data indicate a critical role for stromal MCT4 in supporting the aggressiveness of PDAC tumors.

## DISCUSSION

The presence of abundant desmoplastic stroma represents one of the hallmarks of pancreatic cancer. It is believed that stroma exerts multiple effects on the tumor that contribute to the poor prognosis of pancreatic cancer [22]. In particular the analysis of cultured CAFs suggests that stromal cells provide secreted factors that can support tumor growth, invasion, and metastasis [25, 37, 38]. The data herein suggest that the nature of the stromal environment is complex and has disparate effects on tumor biology. Notably, not all stroma exhibits elevated levels of CAIX and MCT4, and only this form of stroma is associated with particularly poor prognosis. These findings agree well with an emerging literature that PDAC stroma delineates different subtypes of disease [19], and suggest that the metabolic features of the surrounding stroma could be a key determinant of tumor aggressiveness.

While tumor metabolism has been extensively studied, the metabolic features of the tumor microenvironment are less known. In the context of PDAC, where neoplastic cells can represent as little as 10% of tumor volume, microenvironment would be anticipated to have a significant impact on tumor metabolism. It has been shown that glycolytic subtypes of PDAC express high-levels of MCT4 and are dependent on this transporter for maintenance of glycolysis and survival [18]. Surprisingly stromal cells can express exceedingly high levels of MCT4, but remain independent of MCT4 for viability. This finding is consistent with the equally surprising observation that stromal cells do not require glucose for viability, but are dependent on glutamine. Glutamine is the principle fuel for CAF's TCA through a GLUD1 mediated utilization of glutamine. This metabolic adaptation is distinct from the GOT1 mediated pathway that is required to support growth of PDAC cells [14]. Consistent with these findings, inhibitors of GLS1 and GLUD1 restricted the growth of stromal cells. These data suggest that targeting glutamine-dependent metabolic processes may have significant impact on desmoplastic stroma.

There are multiple mechanisms through which a stromal compartment with high-levels of MCT4 and CAIX could impact on tumor biology. Presumably, these markers could simply reflect a hypoxic tumor environment [39, 40]. However, our data with isolated CAFs suggest that the expression of MCT4 while driven by HIF1α, is in fact an epigenetic state that is not simply reflective of hypoxia. Notably HIF1α levels remain exceedingly high in cultures with ample oxygen. Additionally, the elevated HIF1α in CAFs was not reflective of disruption of oxygen sensing and protein stabilization as observed with loss of VHL [41]. Therefore, the basis of high-levels of HIF1α in CAFs remains under study. Importantly, not all cultured PDAC CAFs exhibited high HIF1α and MCT4 levels nor does all tumor stroma exhibit high levels of CAIX and MCT4; therefore indicating the presence of stromal diversity.

High levels of HIF1α enhance glycolytic metabolism and lead to the generation of excess lactate [42]. In this context, acidic pH is known to contribute to tumor cell invasiveness and could be an important contributing factor for the poor prognosis observed in MCT4-high PDAC cases [43]. However, another possibility is that CAFs metabolism provides metabolic intermediates that can be employed by the tumor. Using metabolite tracing we could identify metabolites that were selectively generated from glucose or glutamine metabolism within the CAFs and secreted into media. Whether these metabolites fuel tumorigenesis and progression in the patient is unclear. Additionally, CAFs secrete proteins that could be employed by the tumor cells by macro-pinocytosis [15]. MCT4 knockdown in CAFs selectively impinged on the extracellular production of 3-carbon metabolites and acidification (i.e. ECAR). Since it has been hypothesized that acidic pH promotes local invasive growth and metastasis, this could represent a mechanism via which MCT4-high CAFs contributed to metastatic spread in examined models and particularly poor prognosis in clinical PDAC cases.

Several studies have suggested that targeting PDAC microenvironment could represent an important target for therapeutic interventions. Based on the premise that dense fibrotic and vasculature-poor stroma may provide a barrier to effective drug delivery, strategies targeting the PDAC-associated fibroblasts using hedgehog pathway inhibitors were proposed [24]. However, hedgehog inhibitors failed in preliminary clinical studies, and subsequent studies in mouse models showed that sonic hedgehog genetic deletion or chronic treatment with hedgehog inhibitors resulted in formation of poorly differentiated tumors and reduced survival [27, 28]. These data suggested that stroma, at least in some instances, can play a restrictive role in tumorigenesis. The data herein suggest that by selectively abrogating metabolic features of stroma, it may be possible to specifically limit tumor growth and aggressiveness while maintaining the restrictive role of the stroma on tumor biology. Consistent with this supposition tumors devoid of MCT4 and CAIX in the stroma have a better prognosis in the investigated cohort.

Together the studies here delineate core features of PDAC stroma and patient-derived CAFs that provide insights into features of disease and potential metabolic vulnerabilities.

## MATERIALS AND METHODS

### Cell culture

The established cell lines were cultured as we have previously described [18]. CAFs cultures were established by serially trypsinizing pancreatic tumor specimens and isolating single cells from the tissue. Fibroblastic cultures were confirmed by the presence of vimentin and lack of cytokerain staining. CAFs were cultured in DMEM supplemented with 10% fetal bovine serum with antibiotic and antimycotic.

### Reagents

Cycloheximide (Sigma, 01810-G), MG132 (Selleck, 50-833-9), DMOG (Frontier Scientific, D1070), BPTES (Sigma, SML0601), EGCG (Sigma, 03970590-10MG), AOA (Sigma, C13408-1G), L-Glutamic acid (Sigma, RES5063G-A701X), AZD6244 (Selleck Chem, S1008) were obtained from standard vendors. Universally labeled L-Glutamine (U-13C5) (Cambridge Isotope Laboratories, CLM-1822-H-0.1) and D-Glucose (U-13C6) (Cambridge Isotope Laboratories, CLM1396-1) were obtained from Cambridge Isotope Laboratories. Cell Titer-Glo Luminescent Cell viability assay (Catalog No. G7572) was purchased from Promega Corporation (Madison, WI).

### Antibodies

The following antibodies were obtained from the indicated vendors: MCT4 (Santa Cruz, SC-50329, anti rabbit), MCT1 (Santa Cruz, SC-365501, anti mouse), HIF1α (Novus Biological, NB100-654, anti rabbit), CAIX (Novus Biological, NB100-417, anti rabbit), CD147(R&D system, MAB-972, anti mouse), GAPDH (Santa Cruz, SC25778, anti rabbit), GLUD1 (Novus Biological, NB600-853, anti rabbit), GOT1 (Novus Biological, NBP1-54778, anti rabbit), ALT (Abnova, H00002875-M02A, anti mouse), VDAC (Abcam, ab34726, anti rabbit), β-tubulin (Santa Cruz, SC-9104, anti rabbit), Tom20 (Santa Cruz, SC-17764, anti mouse).

### Study population and tissue microarray construction

Specimens were obtained from 203 largely consecutive patients that underwent resection for pancreatic ductal adenocarcinoma, as was approved by the Institutional Review Board. Clinical and treatment information was extracted by chart review. Supplemental Table 1 contains descriptive statistics (age, tumor size, histologic grade, lymph-node status, stage, vital status, and treatment information). The tumor tissue-microarrays (TMAs) were constructed using a tissue arrayer (Veridiam). TMA sections of 4 μm were used for immunohistochemistry.

### Immunohistochemical staining

CAIX expression levels were assessed using mouse monoclonal anti-CAIX antibody (clone MRQ-54, Cell Marque, Rocklin, CA) at a dilution of 1:500. Vimentin and TP53 expression levels were detected using pre-diluted, anti-vimentin mouse monoclonal and anti-TP53 mouse monoclonal antibodies, respectively (clones V9, DO-7, Ventana, Tucson, AZ). MCT4 expression levels were assessed using rabbit, polyclonal anti-MCT4 antibody diluted at 1:250 (clone H-90, Santa Cruz Biotechnology, Santa Cruz, CA). CD34 expression was detected using pre-diluted mouse, monoclonal anti-CD34 antibody (clone QBEnd/10, Ventana, Tucson, AZ). Immunohistochemical stains were performed using the Dako automated stainer. To evaluate CAIX, vimentin and TP53 expression in tumor epithelial cells, a quantitative scoring system was used based on percentage of positively staining cells. Specimens with 0-5% tumor epithelial cells staining for CAIX were considered negative and those with 6-100% CAIX staining were considered positive, in keeping with previously published criteria [44]. Specimens with any tumor cells staining for Vimentin (>0%) were considered positive. TP53 expression in the specimens was denoted as positive if there was >50% of tumor epithelial cell staining, and negative if staining equaled to or was present in less than 50% of tumor cells [45]. MCT4 expression in tumor epithelial cells was scored semi-quantitatively as follows: 0, 0% immuno-reactive cells; 1, < 5% immuno-reactive cells; 2, 5-50% immune-reactive cells; and 3, >50% immuno-reactive cells. Similarly, intensity of staining was evaluated semi-quantitatively on a scale 0-3, with 0 representing negative, 1, weak; 2, moderate and 3, strong staining. A final score was then calculated by multiplying the two individual values in order to reflect both the percent of immuno-reactive cells and staining intensity, as previously described. For survival analyses, epithelial MCT4 expression was categorized as positive for specimens with a score of 6-9, and categorized as negative for specimens with a score of 0-5.

### Stromal markers scoring

Stromal CAIX was scored categorically as negative (0, no staining) or positive (weak or strong staining). Stromal MCT4 staining was scored semi-quantitatively as negative (0, no staining), weak (1, either diffuse weak staining or strong staining in less than 30% of stromal cells) or strong (2, defined as strong staining of 30% or more of the stromal cells) [46]. For survival analyses, stromal MCT4 expression was dichotomized for specimens with strong staining as positive, and negative for specimens with none or weak staining.

### Microvessel density and stromal volume scoring

Digital pathologic assessment of blood vessels was performed employing microvessel density algorithm (Aperio, Leica Biosystems, Nussloch, GmbH). The CD34 stained slides were scanned on an Aperio ScanScope. The default algorithm parameters were adjusted with the assistance of the Aperio MVA expert representative. The resulting values were stratified around the median into low and high groups, where low microvessel density ranged from 1.03 vessels/mm^2^ to 6.16 vessels/mm^2^ and high microvessel density from 6.2 vessels/mm^2^ to 38 vessels/mm^2^. The percentage of stromal volume was determined on H&E stained whole tumor sections corresponding to blocks used for tissue microarray construction. Visual estimation of the percentage of stromal cells compared to tumor cells was made in one 10X magnification field. Stromal areas were only evaluated when surrounded by tumor cells in all directions. The median value for stromal volume (50%) was used to classify patients into two groups, low and high stromal volume as has been previously published [47].

### Statistical analysis

To determine the clinical significance of biomarker status, we used the endpoint of overall survival. Biomarkers were evaluated for their association with survival through Kaplan-Meier analysis. Univariate and multivariate analysis were performed using the Cox regression method. Correlation between biomarkers was established using Spearman Rank correlation method. Unsupervised, random forest clustering was performed on the biomarkers panel. All analyses were performed using the survival package in R version 3.1.1; heatmaps were created using heatmap.2 in R.

### Plasmids and RNAi

The following RNAi were employed, siMCT4 (Santa Cruz, SC-45892), HIF1α(Sigma, NM_181054). The shRNA against MCT4 was introduced by lenti-viral transduction as previously published [18].

### Immunoblotting

Cells were trypsinized and washed twice with cold PBS. Harvested samples were lysed in ice-cold RIPA lysis buffer (1% NP-40, 0.1% SDS, 0.25% sodium deoxycholate, 150 mmol/L NaCl and 50 mmol/L Tris-HCl (pH 7.4), 1mmol/L EDTA) containing both protease inhibitor cocktail (Roche) and phosphatase inhibitors (Roche). The total protein concentration was determined using a Bio-Rad protein assay reagent (Bio-Rad). Equivalent amounts of proteins were separated by10% or 12% SDS-PAGE and transferred to PVDF membranes (Millipore, IPVH00010). After being blocked in Tris buffered saline (TBS) containing 5% non-fat milk, the membranes were incubated with primary antibodies at a dilution of 1:1000 at 4^o^ C for overnight and then with HRP conjugated anti-mouse or anti-rabbit antibody (Jackson Immunoresearch) at a dilution of 1:5000 at room temperature for 1 hour. Signals were detected on X-ray film using the ECL detection reagent (GE health life science). Equal protein loading was assessed by the GAPDH or Actin.

### Transfection of RNAi

Transfection of siRNA was carried out with Lipofectamine RNAiMAX transfection reagent (Invitrogen, 13778-150) according to the procedure recommended by the manufacturer. Seventy-two hours after transfection with siRNA at various conditions, the cells were harvested for western blot or analyzed on a microplate reader (BioTek) for cell viability assay.

### Metabolomics and mass isotope labeling

For analysis of intracellular metabolites by LC/MS, 10mM of Glucose or 4mM of Glutamine in the DMEM media were replaced either by U^13^C-Glucose or U^13^C-Glutamine. 3 × 10^5^ CAF cells were incubated with isotope labeled nutrient containing media for 6 and 12 hours, for U^13^C-Glucose and U^13^C-Glutamine labeling respectively. 20μl of media samples in each plate were snap frozen for metabolite profiling of media. The preparation of lysates and analysis of the metabolite levels was as previously published [48].

### Cell invasion assay

The invasive behaviors of the Capan2 cells were tested using a transmembrane invasion assay (0.8μm pore size, Costar). CAF were transfected with either siMCT4 RNA or siHIF1α RNA 48 hours prior. Capan2 cells (2.0×10^4^) were plated into the upper chamber and 1×10^4^ of CAF were plated in the bottom wells. Capan2 cells were allowed to invade across the transmembrane for 24 hours at 37°C in 5% CO_2_. After incubation, cells were removed from the upper surface of the filter by scraping with a cotton swab. The invaded cells that adhered to the bottom of the membrane were stained with a crystal violet solution. The number of cells that invaded the membrane was determined by counting the cell number of three randomly selected high-power fields.

### HIF1α stability assays

3 × 10^5^ cells were seeded on 6cm plates. After 24 hours, later, cells were treated with 100μg/ml of cyclohexamide to inhibit protein synthesis. Either MG132 (50μM) or DMOG (50μM) were employed to stabilize HIF1α. Samples were harvested at the indicated time points and lysed in RIPA buffer, then subjected to SDS PAGE and western blot.

## SUPPLEMENTARY MATERIAL


