# Supplementary material for: Unique metabolic features of pancreatic cancer stroma: relevance to the tumor compartment, prognosis, and invasive potential
Source: Oncotarget. 2016 Sep 7;7(48):78396–411. doi: 10.18632/oncotarget.11893 (PMC5346648; doi:10.18632/oncotarget.11893)
Supplement: Supplementary file 1 [file oncotarget-07-78396-s001.pdf]

Unique metabolic features of pancreatic cancer stroma: relevance to the tumor compartment, prognosis, and invasive potential

Supplementary Material

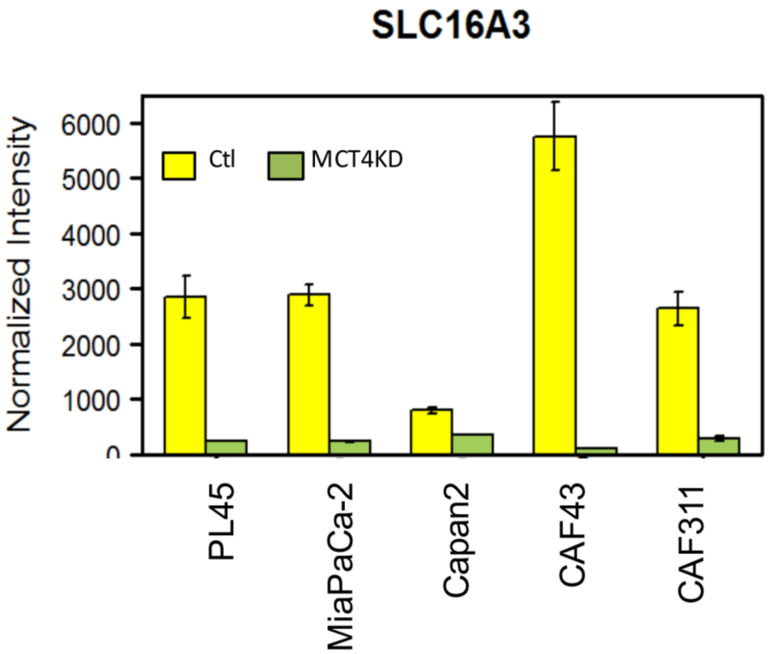

Figure S1: Gene expression analysis of the SLC16A3 gene in parental cell models and CAFs with the knockdown of MCT4 by RNAi.

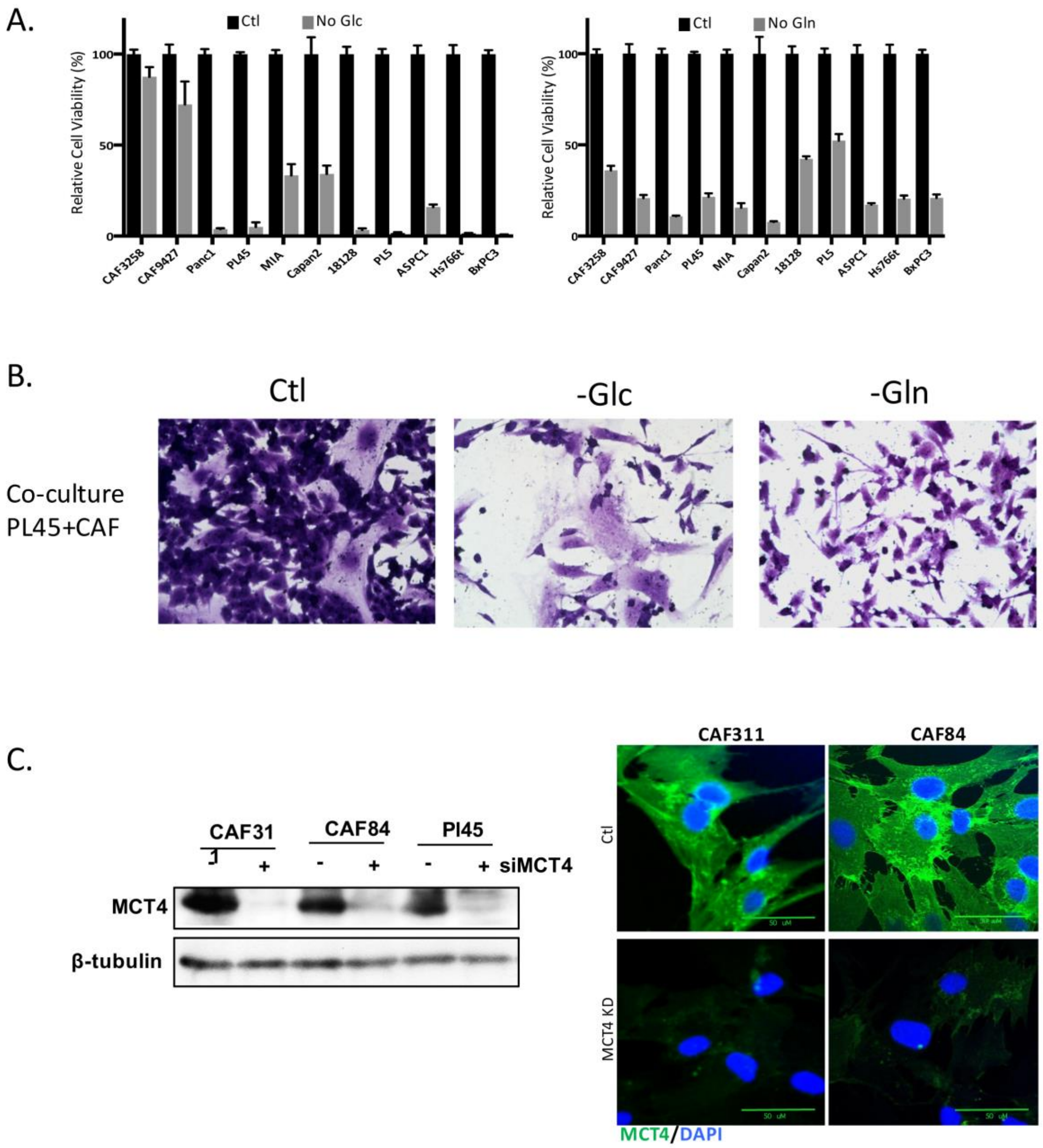

Figure S2: (A) The impact of the withdrawal of glucose or glutamine on the viability of a number of cancer cell lines and CAF cultures was determined. (B) Co-cultures of PL45 cells with CAFs were grown in standard media, or shifted to media lacking glucose or glutamine. Surviving cells were visualized by crystal violet staining. (C) Immunoblot and immunofluorescence staining showing the knockdown of MCT4 in the indicated CAFs and PDAC cell lines.

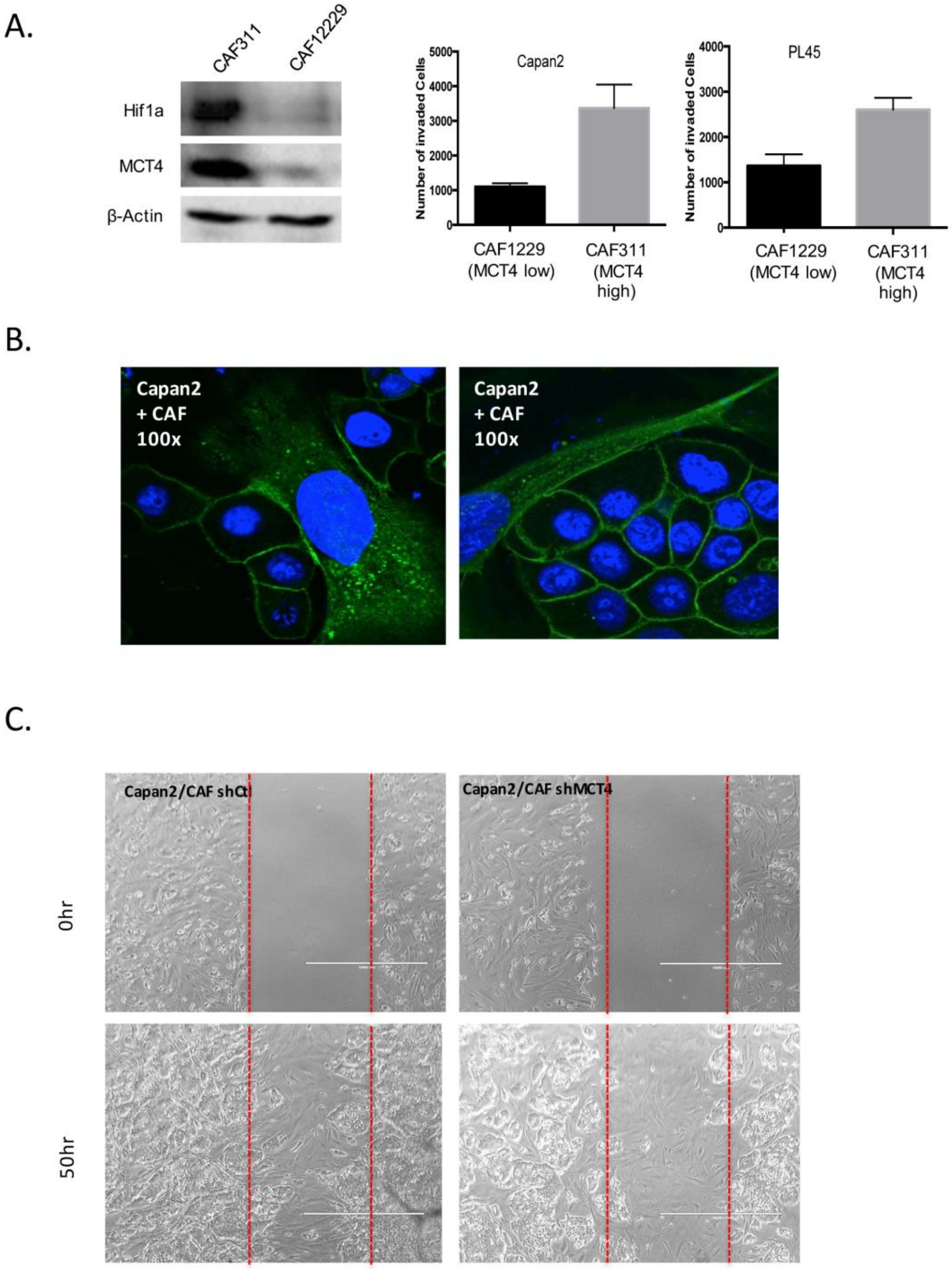

Figure S3: (A) Differential levels of HIF1a and MCT4 in different CAFs cultures. Invasive potential was quantified by Boyden chambers assays with the indicated CAFs cultures. Invasion is associated with the presence of HIF1a and MCT4 in the CAFs cultures using two different PDAC cell lines. (B) Staining of MCT4 in co-cultures of Capan2 and CAFs (C) Wound healing assay demonstrating the invasion of tumor cells into the wounded region and association with HIF1a /MCT4 status.

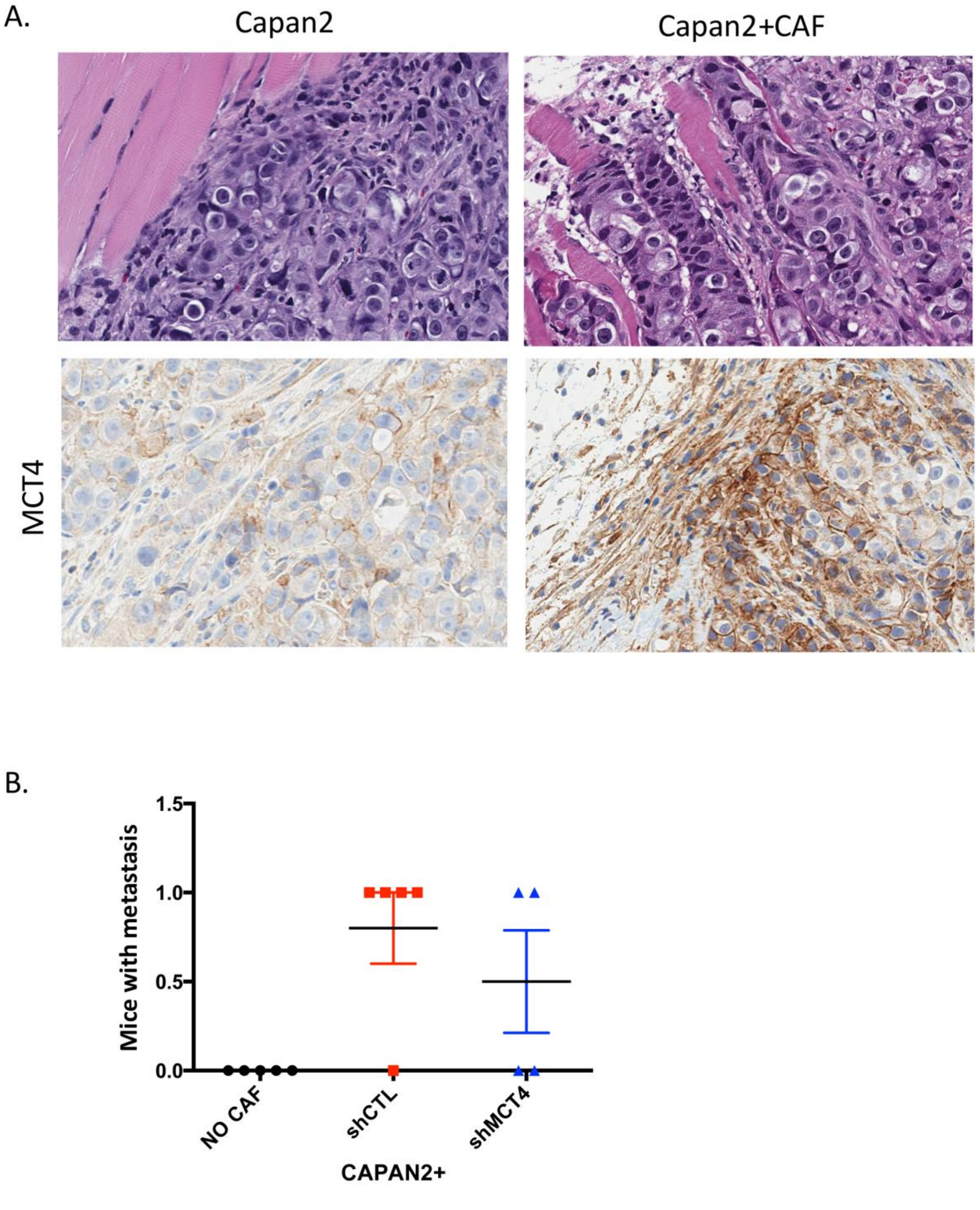

Figure S4: (A) Representative hematoxylin and eosin stained sections of subcutaneous xenografts in the absence or presence of CAFs. Note the invasion into the muscle tissue of the mouse in the presence of CAFs. (B) Quantitation of metastasis from orthotopically injected Capan2 cells in the presence of the indicated CAFs.

Supplementary Tables

1

PDA cohort demographics

Characteristics	No of patients (%)
**Age (years)**<br>Median (range)	203<br>65 (33-84)
**Gender**<br>Male<br>Female	 112 (55)<br>91 (45)
**Tumor size (cm)**<br>0-2<br>3-4<br>>4<br>Unknown	 39 (19)<br>111 (55)<br>45 (22)<br>8 (4)
**Node involvement**<br>Positive<br>Negative<br>Unknown	 127 (62)<br>75 (37)<br>1 (1)
**TNM Stage**<br>Ia<br>Ib<br>IIa<br>IIb<br>III<br>IV<br>Unknown	 12 (6)<br>16 (8)<br>30 (15)<br>130 (64)<br>10 (5)<br>3 (1)<br>2 (1)
**Treatment**<br>Chemotherapy<br>No chemotherapy<br>Unknown	 166 (78)<br>27 (19)<br>10 (3)
**Vital Status**<br>Alive<br>Dead	 114 (55)<br>89 (44)

2

Univariate Analysis

	P-value	Hazard Ratio	95% CI
**Grade**			
3 vs. 1 and 2	0.0026	1.9946	1.272 – 3.127
**Node Status**			
Positive vs. Negative	0.00013	2.4655	1.532 – 3.969
**CAIXtumor**			
Positive vs. Negative	0.4717	0.8222	0.4821 – 1.402
**CAIXstroma**			
Positive vs. Negative	0.0329	1.7546	1.04 – 2.961
**p53**			
Positive vs. Negative	0.0756	1.5354	0.9534 – 2.473
**Vimentin**			
Positive vs. Negative	0.0052	1.8863	1.2 – 2.965
**Microvessel Density**			
High vs. Low	0.02253	1.6878	1.071 – 2.659
**Stromal volume**			
High vs. Low	0.7026	1.0881	0.7252 – 1.73
**MCT4tumor**			
Positive vs. Negative	4.95E-05	2.5918	1.61 – 4.173
**MCT4stroma**			
Positive vs. Negative	2.46E-05	2.585	1.637 – 4.083

3

Correlation Table

	CAIXtumor	CAIXstroma	p53	Vimentin	MVD	Stromal Volume	MCT4tumor	MCT4stroma	Grade
**CAIXtumor**		0.085<br>(0.2673)	-0.143<br>(0.0595)	-0.169<br>(0.0286)	-0.051<br>(0.4903)	-0.089<br>(0.2524)	-0.033<br>(0.6663)	-0.054<br>(0.4723)	-0.085<br>(0.2621)
**CAIXstroma**	0.085<br>(0.2673)		0.165<br>(0.0315)	0.215<br>(0.0057)	0.054<br>(0.4686)	-0.091<br>(0.2491)	0.290<br>(0.0001)	0.292<br>(8.622e-05)	0.089<br>(0.2428)
**p53**	-0.143<br>(0.0595)	0.165<br>(0.0315)		0.200<br>(0.0066)	0.089<br>(0.2102)	0.049<br>(0.5111)	0.162<br>(0.0250)	0.319<br>(1.429e-06)	0.330<br>(2.925e-06)
**Vimentin**	-0.169<br>(0.0286)	0.215<br>(0.0057)	0.200<br>(0.0066)		0.074<br>(0.3091)	-0.114<br>(0.1353)	0.389<br>(4.821e-08)	0.462<br>(3.657e-11)	0.323<br>(7.454e-06)
**MVD**	-0.051<br>(0.4903)	0.054<br>(0.4686)	0.089<br>(0.2102)	0.074<br>(0.3091)		-0.057<br>(0.4166)	0.132<br>(0.0643)	0.062<br>(0.3811)	0.069<br>(0.317)
**Stromal Volume**	-0.089<br>(0.2524)	-0.091<br>(0.2491)	0.049<br>(0.5111)	-0.114<br>(0.1353)	-0.057<br>(0.4166)		0.077<br>(0.3040)	-0.104<br>(0.1587)	-0.217<br>(0.0024)
**MCT4tumor**	-0.033<br>(0.6663)	0.290<br>(0.0001)	0.162<br>(0.0250)	0.389<br>(4.821e-08)	0.132<br>(0.0643)	0.077<br>(0.3040)		0.351<br>(5.811e-07)	0.275<br>(0.0001)
**MCT4stroma**	-0.054<br>(0.4723)	0.292<br>(8.622e-05)	0.319<br>(1.429e-06)	0.462<br>(3.657e-11)	0.062<br>(0.3811)	-0.104<br>(0.1587)	0.351<br>(5.811e-07)		0.336<br>(1.429e-06)
**Grade**	-0.085<br>(0.2621)	0.089<br>(0.2428)	0.330<br>(2.925e-06)	0.323<br>(7.454e-06)	0.069<br>(0.317)	-0.217<br>(0.0024)	0.275<br>(0.0001)	0.336<br>(1.429e-06)	
